# The Distribution of Microplastic Pollution and Ecological Risk Assessment of Jingpo Lake—The World’s Second Largest High-Mountain Barrier Lake

**DOI:** 10.3390/biology14020201

**Published:** 2025-02-14

**Authors:** Haitao Wang, Chen Zhao, Tangbin Huo

**Affiliations:** 1 Heilongjiang River Fisheries Research Institute, Chinese Academy of Fishery Sciences, Harbin 150070, China; zhaochen@hrfri.ac.cn; 2 Heilongjiang River Basin Fishery Ecological Environment Monitoring Center, Ministry of Agriculture and Rural Affairs, Harbin 150070, China

**Keywords:** microplastic, surface water, sediment, fish, ecological risk

## Abstract

To investigate the influence of factors such as tourism, agriculture, and population density on the presence of microplastic (MP) content in aquatic environments and their associated ecological risks, Jingpo Lake, a remote high-mountain lake situated away from urban areas, was selected as the research subject. This study examined the abundance, types, sizes, colors, and polymer compositions of MPs within the water body, fish, and sediments. By considering variables, including fishing practices, agricultural activities, population dynamics, and vegetation cover, an analysis was conducted to unravel the spatial and temporal distribution of MPs concerning human activities, ultimately leading to an assessment of the ecological risks posed by MP pollution. The findings revealed that the average abundance of MPs in the lake’s surface water was recorded as (304.8 ± 170.5) n/m^3^, while in the sediments, it averaged (162.0 ± 57.45) n/kg. Inside the digestive tracts of fish, the MP abundance was measured at 11.4 ± 5.4 n/ind. The contamination of MPs within the aquatic environment of Jingpo Lake was found to be relatively minimal. Variations in MP loads across time and space were observed, with MPs predominantly falling within the size range of small planktonic organisms (50–1000 μm). Additionally, the prevalent colors of MPs in the water samples were white or transparent, constituting approximately 55.65% of the entire MP composition. Subsequently, they were black, red, and blue. This colors distribution were consistent across MPs extracted from fish and sediment samples. The chemical compositions of the MPs predominantly comprised PE (31.83%) and PS (25.48%), followed by PP (17.56%), PA (11.84%), PET (6.71%), EVA (4.56%), and PC (2.03%). Regarding the seasonal aspect, MP concentrations were highest during summer (46.68%), followed by spring (36.75%) and autumn (16.56%). The spatial distribution of MPs within Jingpo Lake’s water body, fish, and sediments was notably influenced by human activities, as confirmed by Pearson correlation coefficients. A strong association was observed between MP levels and water quality indicators such as ammonium nitrogen (NH_4_-N), total phosphorus (TP), and chlorophyll-a (Chla), suggesting that human-related pollution contributed significantly to MP contamination. The diversity assessment of MP pollutants exhibited the highest variability in chemical composition (1.23 to 1.79) using the Shannon–Wiener Index. Subsequently, the diversity of colors ranged from 0.59 to 1.54, shape diversity from 0.78 to 1.30, seasonal diversity from 0.83 to 1.10, and size diversity from 0.44 to 1.01. The assessment results of ecological risk highlighted that the risk categories for MPs within the surface water, fish, and sediments of Jingpo Lake were categorized as I for the PHI and PLI and as “Minor” for the PERI. These relatively low-risk values were attributed to the predominantly low toxicity of the distributed MPs within the Jingpo Lake basin. Moreover, the results of the risk assessment were found to be interconnected with the distribution of the local population and agricultural activities around the sampling sections. Usage patterns of coastal land and population density were recognized as influential factors affecting MP loads within the water body, sediments, fish, and other components of the lake ecosystem.

## Simple Summary

In order to understand the impact of population, tourism, and agriculture on microplastic pollution in freshwater lakes, we conducted our first study on the loading and ecological risks of microplastics in Jingpo Lake The abundance of microplastics in the surface water, sediment, and fish digestive tract of Jingpo Lake belongs to a moderate range, and its spatial and temporal distribution is influenced by factors such as human settlements, agricultural practices, and tourism activities. The value of the microplastic risk assessment index in Jingpo Lake is relatively low. This study contributes to a more comprehensive understanding of the global distribution of plastic pollution.

## 1. Introduction

Microplastics (MPs), defined as plastic particles ranging in size from 0 to 5 mm, have emerged as a subject of significant concern due to their worldwide distribution and potential toxicity in the environment. The production and use of plastic products, daily life and industrial emissions, agricultural production, marine aquaculture and shipping, and the development of tourism are all human activities that increase microplastic pollution in water bodies [1,2,3]. The increasing prevalence of MP pollution reflects the exponential production growth, with projections indicating an annual output exceeding 1.8 billion tons by 2050 [4]. Presently, the contamination of aquatic ecosystems with MPs has garnered global attention as an environmental issue of utmost importance. MPs, propelled by factors such as surface runoff, wind dispersion, and precipitation, ultimately find their way into water environments, effectively turning these habitats into reservoirs for MPs. Research efforts on MP pollution in freshwater ecosystems have primarily centered around urban-adjacent rivers and lakes [5,6]. However, studies focusing on water bodies situated far from human settlements and industrial zones have been relatively limited. Lakes and reservoirs in urban centers or their peripheries are often located near residential and industrial areas. As a result, they are more susceptible to MP pollution originating from sewage discharge, surface runoff, and both wet and dry deposition. Moreover, these water bodies tend to exhibit elevated MP content due to the presence of industrial and municipal wastewater, making them less ideal for discerning patterns of agricultural MP pollution and assessing the associated risks in rural regions that are sparsely populated [7,8].With their substantial surface area relative to their volume and inherent hydrophobic characteristics, MPs serve as carriers for pesticides, organic pollutants, heavy metals, and pathogens. This accumulation of contaminants can lead to adverse impacts on human health [9]. Agricultural regions are recognized as significant sources of pollutants, particularly pesticides. Consequently, they play a pivotal role in the pollution load. These contaminants adhere to MPs that arise from the degradation of agricultural films, potentially causing substantial harm to the environment, aquatic organisms, and even human well-being [10]. In light of these considerations, a comprehensive and systematic examination and evaluation of MP pollution across non-urban or suburban environments, encompassing rivers, lakes, sediments, and fish, assume critical importance for preserving the health of aquatic ecosystems and the assurance of water security [8,11].

Jingpo Lake graces the southeastern expanse of Heilongjiang Province, China. The climate is characterized by a semi-humid oceanic temperate monsoon climate. The annual precipitation ranges from 500 to 600 mm. As the world’s second-largest high-mountain barrier lake, it owes its genesis to the confluence of Quaternary volcanic eruptions and the Mudanjiang River’s course, resulting in the obstruction of its bed. This geological occurrence yielded China’s largest high-mountain barrier lake [12]. A dam was constructed at the lake’s outlet, birthing a large reservoir primarily harnessed for hydroelectric power, complemented by ancillary benefits spanning irrigation, urban water supply, and tourism. The abundant water resources and the superior natural environment have fostered a diverse wealth of freshwater fish within the lake’s depths [13]. Historical records detail a spectrum of 52 fish species spanning 12 families, with the Cyprinidae family taking precedence, amounting to 37 species, including their subspecies. Jingpo Lake serves as an invaluable genetic reservoir for economically significant fish species along the middle stretch of the Mudanjiang River [14]. A notable player among these is the Mongolian redfin (*Culter mongolicus*), commonly known as the redtail. Renowned for its tender, delectable flesh, the Mongolian redfin stands as a cornerstone of the lake’s economic fishery, boasting a considerable market value. In recent years, intriguing trends have emerged, characterized by diminished fish size and younger age [15]. Moreover, the sampling process frequently unveils anomalous fish forms. These occurrences may find their origins in environmental shifts, possibly linked to MP pollution and analogous influences. Furthermore, the selection of Jingpo Lake as our research subject stems from two principal attributes [16]. First, its geographical remoteness from urban agglomerations, industrial complexes, agricultural activities, and human settlements engenders an equitable distribution of influencing factors. This contrasts with lakes in close proximity to cities, where distinguishing the distinct contributions of industries, such as agriculture and tourism, to waterborne MP levels becomes challenging. Secondarily, the elongated contours of Jingpo Lake set it apart. Unlike the majority of circular, elliptical, or rectangular lakes, its sampling section in the central area lies proximate to the shoreline. This configuration ensures a comprehensive and thorough dispersion of contaminants. Consequently, this unique morphology presents an ideal focal point for investigating the interplay between human activities and MP pollution across both lakefronts [5,16]. According to the data of the National Bureau of Statistics of China, the number of overseas tourists visiting Jingpo Lake decreased by 94.45% year-on-year. The domestic tourism market also has poor revenue, with a year-on-year decrease of 56.88% in domestic tourism revenue in 2020. As a tourist lake, the pollutant load is usually greatly affected by tourism. After the COVID-19 pandemic, the tourist population decreased, providing a good opportunity for us to investigate the impact of other human activities on the microplastic load of Jingpo Lake.

The objectives of this study are outlined as follows: (1) We analyze the abundance and spatiotemporal distribution of MPs in the surface water, sediments, and digestive tracts of fish in the Jingpo Lake. (2) We investigate the diversity of MP pollution and elucidate its sources. (3) We evaluate the ecological risks associated with MPs in the lake. (4) We explore the impact of factors such as land use patterns, land cover, and population density along the lake shore on the MP load in water, sediments, and fish.

## 2. Materials and Methods

### 2.1. Study Site and Sample Collection

Jingpo Lake (located at 128°46′–129°02′ longitude and 43°46′–44°01′ latitude) graces the southeastern expanse of Heilongjiang Province, China, nestled between the Zhangguangcai Ridge and the Laoyeling Ridge. As the world’s second-largest high-mountain barrier lake, it owes its genesis to the confluence of Quaternary volcanic eruptions and the Mudanjiang River’s course, resulting in the obstruction of its bed. This geological occurrence yielded China’s largest high-mountain barrier lake. Flowing from the Mudanjiang Ridge of Jilin Province’s Changbai Mountains, the Mudanjiang River gushes with a vigorous descent, positioning Jingpo Lake approximately 300 km downstream from its origin, cascading across an impressive vertical gradient of about 750 m. An intricate network of over 30 rivers of varied dimensions converge radially into the lake. The Jingpo Lake stretches a north–south expanse of 45 km, embracing its widest breadth of 6000 m east to west before tapering to a mere 300 m breadth at its most slender. The prevailing width typically oscillates between 500 and 1000 m. The zenith of depth, resting within the lake’s northern sector, descends to a profound 70 m. Encompassing a watery domain spanning 79.3 km^2^, Jingpo Lake embraces an immense reservoir capacity of approximately 1.6 billion m^3^. The Jingpo Lake comprises over 30 rivers of varying sizes, converging in a radial pattern as they flow into the lake. These rivers predominantly take the form of mountain streams characterized by their rapid currents and swift runoff. They exhibit significant sediment-carrying capacity, high annual water flow, pronounced seasonal fluctuations, and an extended period of ice cover. Towards the southern end of Jingpo Lake, it intertwines with the upper reaches of the Mudanjiang River and numerous tributaries.

**Figure 1:**
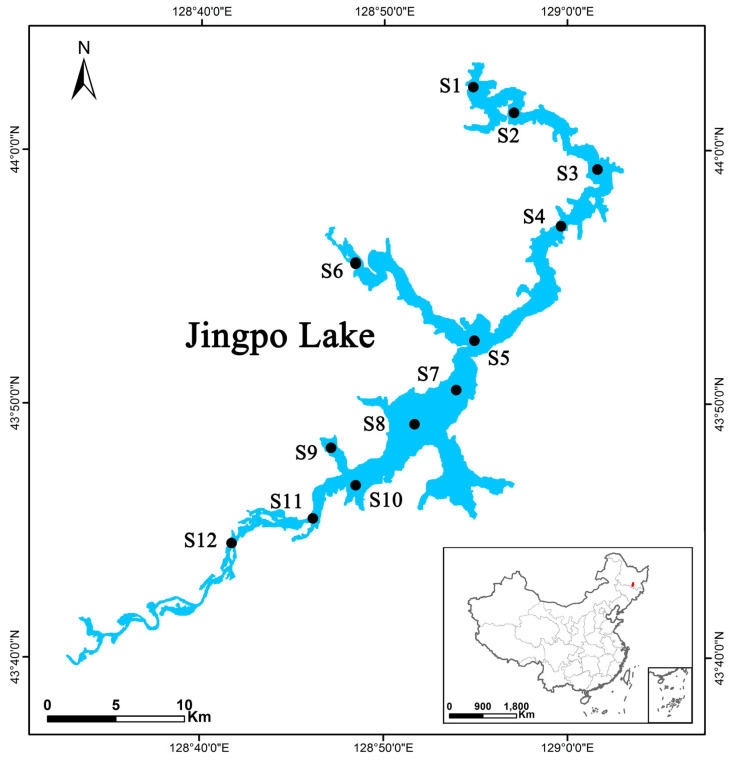
Sampling sections in the Jingpo Lake reservoir. S1–S4 locations are close to settlements including densely populated areas, tourist ports, hotels, and related reception infrastructure, while S2, S3, S10, S11, and S12 are closer to farmland, and other sampling sections are areas with less human activity.

Based on the human activities along the lake shore, including agriculture, tourism, and pastoralism, as well as natural factors such as temperature, precipitation, and monsoons, the sampling for this study was conducted in May (spring), August (summer), and October (autumn) of 2020 to 2023. The selected sampling sections encompassed various lake habitats, including inflow-to-outflow areas, shoreline areas, sub-shoreline areas, and deep-water areas. Diverse levels of vegetation cover, population density, land use types, and substrate characteristics were taken into account when setting up the sampling sections. Additionally, factors related to the morphology of the lake basin, such as the steepness of the lake basin and variations in water depth, were considered when establishing the transects (locations, lengths, and numbers) and stations (density and distance from the shore). Significant fish habitats, including spawning grounds, foraging areas, overwintering sites, and migration pathways, were designated as specific sampling sections [15]. Guided by the principles above and informed by historical surveys, a total of 12 survey stations were strategically positioned across Jingpo Lake (Figure 1). At each sampling section, water, fish digestive tracts, and sediment samples were meticulously collected, with three parallel samples collected for each type.

Surface water samples were meticulously collected in sets of three, with each sample representing a blend of the surface, mid-depth, and lower layers of water. For the surface water samples, three designated sites were chosen within 20 cm of the water’s surface. In locations where water depth exceeded 200 cm, specific sites were identified to collect mid-depth and lower-layer water samples, maintaining a distance of 100 cm from the water surface. To facilitate the collection process, a submersible pump (2400-12AH, Sinleader, Zhejiang Shengshi Yuanlin Technology Co., Ltd., Taizhou, China) was utilized, pumping water for a duration of 30 s at each station. A total of 100 L of water was extracted and then gently filtered through a portable mesh with a pore size of 20 μm, effectively channeling the filtered water into 100 mL sampling bottles. To ensure the preservation, 0.5 mL of formaldehyde fixative was carefully introduced to the samples before they were securely stored in light-shielded refrigeration units operating within the temperature range of 0–4 °C. The samples were promptly transported to the laboratory for subsequent analysis of MPs. When collecting sediment samples from the surface layer (with depths ranging from 0 to 25 cm), a Peterson grab sampler was employed. Approximately 2 cm of the uppermost surface layer was meticulously placed into 1 L amber glass bottles using a stainless steel spoon. For the retrieval of samples from the digestive tracts of aquatic organisms and other biological specimens, a metal spoon was used, and the samples were meticulously sealed within aluminum foil bags. All collected samples were promptly transported to the laboratory and stored under refrigeration until the subsequent phase of MP analysis commenced.

### 2.2. Laboratory Analysis

#### 2.2.1. Pretreatment of Microplastics in Water Samples

Approximately 100 mL of hydrogen peroxide (30%, *V*/*V*) was added to each water sample and thoroughly mixed. The samples were allowed to react at room temperature for 12 h to facilitate the decomposition of organic matter. Tin foil was used to cover the bottle openings to prevent potential MP contamination from the air. Suction filtration was performed using a vacuum pump and glass fiber filter paper with a pore size of 0.45 μm, and the filtered paper was stored in dry petri dishes. Sediment samples were dried in an oven at 60 °C until a constant weight was achieved. A 5 mm stainless steel sieve was used to remove any interfering elements like stones that could affect the experiment. A weighed amount of 100 g of dried sediments was mixed with 200 mL of hydrogen peroxide and 20 mL of ferrous sulfate heptahydrate (0.05 m·L^−1^). The mixture was thoroughly stirred and allowed to react for 12 h at room temperature to break down organic matter. Similar to the water samples, tin foil was used to cover the bottle openings to prevent MP contamination from the air. MP extraction was conducted using the density flotation method. A saturated sodium chloride solution (150 mL) was added and thoroughly mixed, followed by 12 h of settling. Vacuum filtration was carried out using 0.45 μm glass fiber filter paper (Whatman, Maidstone, UK), and the filtered paper was stored in dry petri dishes. In terms of quality control, experimenters wore cotton lab coats and gloves throughout the sampling and laboratory processes. All experimental equipment was thoroughly washed with ultrapure water before use. Samples exposed to the air were covered with tin foil. Control groups were established, and no MPs were found.

#### 2.2.2. Pretreatment of Microplastics in Biological Samples

For the biological samples, a concentrated nitric acid digestion method was employed. The Mongolian redfin (*Culter mongolicus*), which occupies a dominant position in the fish population of Jingpo Lake, was selected as a biological sample. Approximately 5 g of the sample was placed in a 100 mL beaker, and around three times the volume of concentrated nitric acid (68%) was added to the beaker. After allowing the mixture to stand at room temperature for 48 h, it was further heated to 95 °C for 3 h to ensure thorough protein digestion. The solution obtained after digestion was subjected to vacuum filtration using stainless steel filter membranes. The filter membrane was initially rinsed with ultrapure water, and after discarding the solution in the filtration flask, it was thoroughly flushed with tap water. Subsequently, the filter membrane was washed extensively with ethanol. The washed filter membrane was then immersed in an ethanol solution and subjected to ultrasonic treatment to disperse the substances from the filter membrane into the ethanol solution. After removing the filter membrane from the ethanol solution, it was washed multiple times with ethanol before placing the ethanol solution in an oven set at 60 °C for drying.

#### 2.2.3. Pretreatment of Microplastics in Sediment Samples

The sediment samples underwent zinc chloride flotation for processing. Approximately 20 g of dry sediment sample was placed in a 100 mL beaker, and 60 mL of zinc chloride solution with a concentration of 1.7–1.8 kg/L was added. The mixture was thoroughly stirred for about 10 min and left to settle overnight. The suspension was then transferred to another 100 mL beaker, and 60 mL of 30% H_2_O_2_ was introduced to remove organic matter. After thorough mixing, the solution was allowed to settle for 24 h to facilitate the complete reaction between hydrogen peroxide and organic compounds. The resulting supernatant underwent vacuum filtration. If deemed necessary, a steel mesh with a pore size of 500 μm could be initially used to intercept larger particles. The obtained filter membrane was immersed in an ethanol solution and subjected to ultrasonic treatment to ensure the dispersion of substances from the filter membrane into the ethanol solution. After extracting the filter membrane from the ethanol solution, it was washed multiple times with ethanol before placing the ethanol solution in an oven set at 60 °C for drying.

#### 2.2.4. Detection Method of Microplastics in All Samples

Currently, the predominant methods for MP detection include laser infrared spectroscopy, microscopic infrared spectroscopy, Raman spectroscopy, and gas chromatography–mass spectrometry. In this study, tailored to the specific requirements, MP analysis in the samples was conducted using a combination of laser infrared spectroscopy and microscopic infrared spectroscopy. Laser infrared spectroscopy (utilizing the Agilent 8700LDIR laser infrared imaging system, Agilent Technologies, Santa Clara, CA, USA) was applied for detecting MPs below 500 μm, while microscopic infrared spectroscopy was employed for MPs within the range of 500 to 5000 μm. The particle analysis mode was selected, the microplastic infrared spectral library establishment method was chosen, and an automatic testing method was set (matching degree > 0.65).

### 2.3. Quality Control in Experiments

Several quality control procedures were employed during the experiment to mitigate potential sources of contamination. Prior to use, all solvents underwent filtration through 1.2 μm polytetrafluoroethylene membranes. All experimental consumables and equipment were either glass or stainless steel to prevent contamination. Aluminum foil was utilized to cover samples between each step throughout the experiment. During the analysis phase, a 20 L volume of ultrapure water was passed through a 15 μm mesh plankton net, followed by vacuum filtration through a 0.45 μm glass microfiber filter paper as a procedural blank. This was performed to minimize the risk of introducing contamination during the experimental procedures.

### 2.4. Research Methods for the Diversity of MP Pollution

The Shannon–Wiener Index, a commonly used diversity metric, combines both the abundance and evenness of different species or types within a given environment. By employing this index, we can quantitatively assess the level of uncertainty in the structure of the system. In this study, we utilized the Shannon–Wiener Index to calculate the MP diversity at each sampling section within the investigated area.

### 2.5. Research Methods for the Ecological Risk Assessment

When assessing the potential risks of MPs in Jingpo Lake, it is crucial to consider risk evaluation models that incorporate both MP polymer toxicity data and MP abundance.

Polymer hazard index (PHI), pollution load index (PLI), and potential ecological risk index (PERI) models were employed to assess the environmental risks of MPs in water samples.

### 2.6. Statistical Analysis

Data analysis was conducted using the SPSS 29.0 software (IBM Corporation, Armonk, NY, USA). The average abundance of MPs was compared seasonally and spatially using the Kruskal–Wallis test and Mann–Whitney U test, respectively. The correlation between MP abundance in the watershed and various environmental indicators was assessed through Pearson’s correlation coefficient. All results were considered statistically significant if *p* < 0.05.

## 3. Results and Conclusions

### 3.1. MPs Abundance

MPs were detected in both surface water and sediment samples from all sampling sections within Jingpo Lake reservoir. The average MP abundance in the surface water was recorded as 304.8 ± 170.5 n/m^3^ (Table 1). In the East Lake, Hubei, situated within the Yangtze River Basin in China, MP concentrations ranged from 952.38 to 10,285.71 n/m^3^, with an average of 3329.19 ± 2059.26 n/m^3^ [5]. MPs were also identified in the waters of China’s Taihu Lake, with abundance ranging from 1.8 to 18.2 n/L [17]. For Lake Superior, MP abundance in water samples ranged from 9000 to 40,000 n/km^2^ [18]. Lake Huron exhibited MP abundance spanning from 59 to 335,714 n/kg [6]. A study by [19]. demonstrated that within the Laurentian Great Lakes, among the five major lakes, a total of 21 samples were collected from three lakes, exhibiting a wide range of MP counts from 0 to over 450,000 n/km^2^, with an average count density of 43,157 ± 115,519 n/km^2^. In Rewalsar Lake, MPs were identified across all samples, with concentrations of 13–238 n/L and 750 to 3020 n/kg [20].

The average abundance of MPs in sediments was (262.2 ± 143.5) n/kg (Table 1). In sediment samples from East Lake, the MP abundance ranged from 735.43 to 5021.78 n/kg, with an average of 2207.56 ± 1194.04 n/kg and a detection rate of 100% [5]. In Elk Lake, the abundance of MPs was 80 ± 30 n/kg; in White Iron Lake, it was 30 ± 20 n/kg; in Ten Mile Lake, it was 180 ± 130 n/kg; and in Peltier Lake, it was 270 ± 200 n/kg. Among these lakes, the two with less human disturbance, Elk Lake and White Iron Lake, exhibited lower levels of MPs. The two lakes with higher human activity, Ten Mile Lake and Peltier Lake, showed MP concentrations roughly comparable to those in this study. However, it should be noted that different lower thresholds for MP size might affect the observed differences in MP concentrations. In study on Minnesota, a lower threshold of 250 μm was used. Due to biological fouling, smaller plastics are more likely to be deposited in sediments, potentially increasing sediment concentrations significantly [4]. In sediments from Taihu Lake, MP abundance ranged from 460 to 1380 n/kg [17]. For Lake Huron, sediment concentrations ranged from 59 to 335,714 n/kg [6].

The average abundance of MPs in the digestive tracts of fish was 11.4 ± 5.4 n/ind (Table 1). MPs were detected in 44% of white sucker fish, ranging from 0 to 14 n per fish in the upper Thames River, Ontario. The MP content in these fish was similar to that observed in the chosen fish species for this study. MPs were found in 31% of common carp in the upper Thames River, Ontario, ranging from 0 to 128 n/individual [10]. The notably higher content of MPs in the digestive tracts of common carp, compared to the selected Mongolian redfin in our study, can be attributed to variations in individual size, mouth dimensions, and feeding behavior. The carp examined in a research were substantially larger in size and had larger mouths compared to the Mongolian redfin selected for this study. Moreover, the carp were predatory in nature, leading to a heightened uptake of MPs. In Alpine high-mountain lakes, the average concentration of MPs identified in the gastrointestinal tracts (GIT) of Salvelinus fontinalis from the lower lake (0.45 n/g GIT) was significantly higher than those from the upper lake (0.20 n/g GIT). A negative correlation existed between fish size (weight and age) and the MP abundance within the fish GIT, implying that juvenile fish amassed more MPs, possibly due to their elevated consumption rate of prey compared to adult fish. The research findings of some researchers advocate for employing *S. fontinalis* as an indicator of MP pollution in high-mountain lake ecosystems. Additional investigation is necessary to gain a more comprehensive understanding of the sources and repercussions of MPs in these isolated ecosystems [8,21].

In comparison to other watersheds, the abundance of MPs in the water, sediments, and fish digestive tracts of Jingpo Lake is relatively low (Table 1).

**Table 1:** Comparison of MP abundance in Jingpo Lake and other water bodies.

Lakes	Concentration of MPs	Reference
Jingpo lake, China	The average abundance of MPs in water was (304.77 ± 170.5 n/m^3^, (262.2 ± 143.5) n/kg in sediments, and 11.4 ± 5.4 n/individual in fish digestive tracts	This study
Xinghu lake, China	MPs varies during wet and dry seasons: MPs in water is 247 ± 120.6 and 273.1 ± 353.7, MPs in sediments is 4.8 ± 2.2 and 10.1 ± 7.6 n/m^3^	[11]
Lake Huron, Canada	MPs in sediments: 59–335,714 n/kg	[6]
East Lake, China	MPs in water was 3329.19 ± 2059.26 n/m^3^; MPs in sediments: was 2207.56 ± 1194.04 n/kg.	[5]
Taihu Lake, China	MPs in water: 1700–8500 n/ m^3^; MPs in sediments: 460–1380 n/kg	[17]
Lake Manipal, India	MPs in water: 423 (±250) n/m^3^. After the monsoon season, the average (±SD) abundance decreased to 117 (±40 n/m^3^).	[22]
Ox-Bow Lake, Yenagoa, Nigeria	MPs in water: During the dry season, the abundance ranged from 1004 to 8329 n/m^3^, while during the rainy season, it ranged from 201 to 8369 n/m^3^.	[23]
Kodaikanalan Lake (freshwater), Indian	MPs in water: 24,420 ± 32,220 n/m^3^; MPs in sediments: 28.31 ± 5.29 n/kg.	[24]
Chaohu lake, China	MPs in fish: 9.07 ± 5.89 n/ind during the rainy season.	[25]
Poyang Lake, China	MPs in water: 5000–34,000 n/m^3^; MPs in sediments: 54–506 n/kg for sediments; MPs in fish digestive tracts: 0–18 n /ind. for wild crucians (*Carassius auratus*).	[26]
Upper Thames River Ontario, Canada	MPs in fish digestive tracts: MPs were found in 44% of white suckers, ranging from 0 to 14 n/ind, and 31% of common carp, ranging from 0 to 128 n /ind	[10]
Kumaraswamy Lake, Coimbatore, India,	MPs in water: 10,160 ± 3200 n/m^3^ (pre-monsoon), 11,330 ± 5500 n/m^3^ (monsoon), and 8910 ± 570 n/m^3^ (post-monsoon).	[7]
Vellayani Lake, Kerala, India	MPs in water: 41,000 n/m^3^; MPs in sediments: 5.4 n/kg	[9]
74 high-mountain lakes of Sierra Nevada, Spain	MPs in water: 300–21,300 n/m^3^.	[27]
Two high-mountain lakes (Upper Lake Balma and Lower Lake Balma) in the Cottian Alps, Italy	No MPs were found in the water;MPs in sediments: 1.33 ± 0.67 n/m^3^ and 1.75 ± 0.62 n/m^3^ in Lower and Upper Lake Balma; MPs in fish digestive tracts: from the Lower (0.45 n/g GIT) than in those from the Upper Lake (0.20 n/g GIT)	[8]
Lake Superior, Lake Huron, Lake Erie, North America	MPs in water: For Lake Superior, the range was 1277–12,645 n/km^2^; for Lake Huron, it was 0–6541 n/km^2^; and for Lake Erie, it was 4686–466,305 n/km^2^.	[19]
White Iron Lake, USA	MPs in water: 152,000 ± 154,000 n/km^−2^; sediments: 30 ± 20 n/kg	[21]
Ten Mile Lake, USA	MPs in water: 58,000 ± 23,000 n/km^−2^; sediments: 180 ± 130 n/kg	
Peltier Lake, USA	MPs in water: 110,000 ± 58,000 n/km^−2^; sediments: 270 ± 200 n/kg	
Elk Lake, USA	MPs in water: 27,000 ± 16,000 n/km^−2^; MPs in sediments: 80 ± 30 n/kg	

### 3.2. MP Composition and Temporal–Spatial Variations

This study observed the temporal–spatial variations in MP loads, highlighting the crucial need for a comprehensive assessment of MPs. Given the high variability in the physical and ecological characteristics of inland lakes, even within a specific region or basin type, accurately predicting MP loads is challenging. The samples in this study exhibited significant variability in repetitions, underscoring the necessity to consider factors such as size, shape, color, chemical composition, and season when discussing the temporal–spatial distribution patterns of MPs [23].

**Figure 2:**
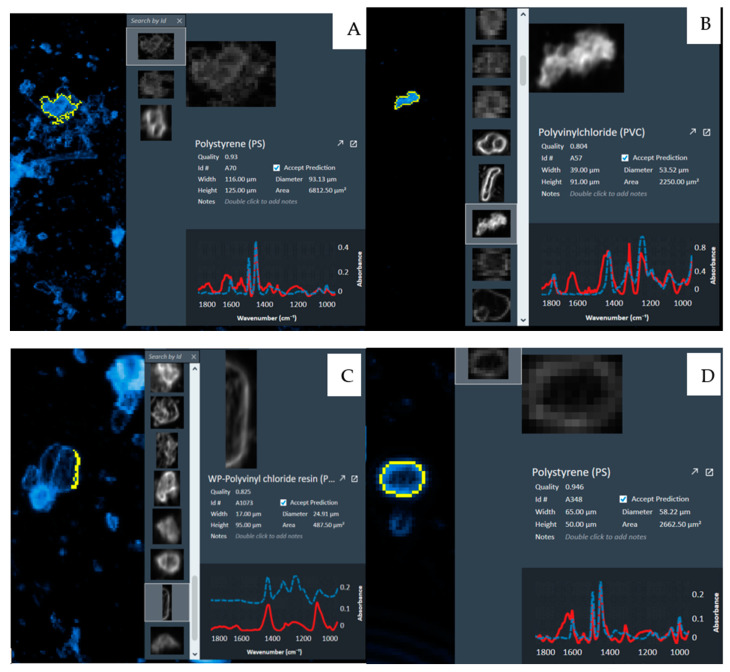
Profile images of typical MPs and occurrence characteristics of MPs in different sampling sections. (**A**): Fragment(PS); (**B**): Film(PVC); (**C**): Fiber(PVC); (**D**): Microsphere(PS). The outline of microplastic properties is surrounded by yellow lines.

When studying planktonic organisms in natural aquatic environments, they are typically classified into three categories based on size: microplankton (<50 μm), such as dinoflagellates and chrysophyceaes; small plankton (50–1000 μm), including diatoms and cyanobacteria; and mesoplankton (1000–5000 μm), such as copepods [28]. The harm of MP pollutants to aquatic organisms mainly arises from the fact that MPs are easily mistaken for food and consequently enter the food chain. Therefore, when researching MPs, it is crucial to categorize and analyze them based on their sizes. In the water samples collected from Jingpo Lake, MPs are predominantly found in the size range corresponding to small plankton (50–1000 μm), exhibiting a proportion similar to that seen in fish and sediment samples (Figure 2). This might be due to the propensity of MPs within this size range to stay in water and sediments, making them more susceptible to ingestion by fish and hence more prevalent in their digestive tracts. Different lower threshold values for MP size could also introduce variations in the observed MP concentrations. In a study by Peter et al. in Minnesota, it was revealed that smaller plastic particles were more likely to accumulate within sediments, suggesting that adopting a smaller value of size threshold could significantly elevate sediment concentrations [21]. In the inflowing rivers to Taihu Lake, the primary MP particle size is <100 μm, while in the lake water and outflowing rivers, the predominant size range of MP particles is 100–200 μm. MPs smaller than 100 μm account for only 28% of the lake’s surface water, yet they significantly increase to 70% in the sediments. This indicates that smaller MPs might have a greater propensity to settle in the lake [17]. In Lake Huron, MP microfibers constitute the major particle, comprising 50% of the total count (adjusted), with an average length of 1.2 mm. Fragments make up 30% of the total count, with average sizes ranging from 53 to 500 μm [6]. In Finnish lakes, plastic fibers detected were larger than fragments in both water and sediment samples, with fiber sizes ranging from 1100 ± 230 μm to 1300 ± 120 μm. The size of plastic fragment detected in water samples were 430 ± 49 μm, while in sediment samples, they were 410 ± 38 μm. Nevertheless, no significant differences in MP size were observed between sampling sections [29]. According to a study in 2013, the average abundance of MPs in the Laurentian Great Lakes was 43,157 n/km^2^, with particles in the size range of 0.355–0.999 mm, accounting for 81% of the total particle count [19].

**Figure 3:**
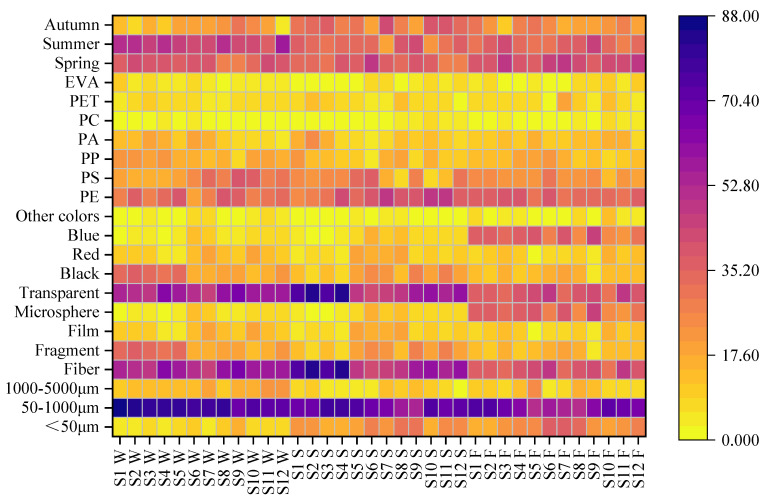
MP types and temporal–spatial distribution in Jingpo Lake. S1W–S12W: MPs in water; S1S–S12S: MPs in sediments; S1F–S12F: MPs in fish digestive tracts.

Four different shapes of microplastics were detected in our research works; they are fragments, films, fiber, and microspheres (Figure 2). Two sampling methods (Mann–Whitney U test, *p* > 0.05) and the composition percentage of each shape for all sampling sections via each method indicated no significant differences (Kruskal–Wallis test, *p* > 0.05). In the samples for Taihu Lake, fibers constitute the majority of MPs at 55.65% of the total count, followed by fragments (24.00%), films (11.57%), and microspheres (5.75%) (Figure 3). The distribution of MP shapes in fish and sediment samples is similar to that of water samples. These observations are linked to coastal human activities: fibrous MPs commonly result from the weathering and breakdown of human-made items like clothing, ropes, and fishing nets. Fragmented MPs predominantly originate from the degradation of plastic waste, including construction materials and plastic bottles. Film-shaped MPs mainly arise from the disintegration of plastic products like shopping bags and agricultural films. Microspheres primarily come from consumer goods such as cosmetics and personal care items. In areas with limited coastal industrialization, everyday products become a significant source of MPs. In both the US Great Lakes and urban lakes in China, around half of the detected MPs are in the form of fibers. Regarding the MP forms found in Lake Superior’s sediments and water samples, the percentage of fibers in water samples (70%) is slightly higher than in sediment samples (52%). Among sediment samples, MPs primarily exist in fiber form (52%), followed by films (28%) and then fragments (18%) [18]. In Lake Huron, North America, the majority of particles examined are microfibers (n = 257), followed by fragments (n = 88), while microspheres and films make up 13% and 7% of the remaining distribution, respectively. Only three sampling sections contained microspheres (n = 7) [6]. In Finnish lakes, MP fibers in sediments (70%) are typically more abundant than those in water samples (40%). In contrast, regardless of the sample matrix, over 80% of MPs in blanks are fragments. Therefore, the concentration of MP fragments reported in both sample types is more likely to be influenced by contamination compared to the concentration of MP fibers. Differences in the relative proportion of MP shapes detected may arise from varying types of MP sources around the study locations, such as sewage treatment plants, littering, and fishing activities. However, due to the sampling, pre-processing, and identification methods employed, certain limitations in detecting specific MPs may exist, potentially contributing to variations [29]. In Lake Rawaal, the majority of MPs are fragments (82%). The colors of MPs are diverse, with white/transparent and black MPs being common. Polypropylene is the predominant type of MPs in Lake Rawaal (40–74%) [16].

Microplastic colors were detected separately using a stereomicroscope in our research work. Plastic products used in various industries often come in different types. For example, agricultural plastic films are typically transparent or white, while colored plastics such as black, blue, and yellow are commonly used in clothing. Plastic bags are usually white or transparent, but some regions and sectors also utilize blue, green, red, and other colors. For instance, in many of China’s seafood markets, black plastic bags are often preferred. Judging the source or use of microplastics solely based on their color is clearly too vague, but this method provides us with an idea before more precise traceability techniques are developed, but it still needs to be improved. Therefore, analyzing the colors of MPs can greatly assist in delving deeper into their sources and associated risks. MPs in water samples of Jingpo Lake are predominantly white or transparent, accounting for 55.65% of all MPs. Following these are black, red, and blue colors. The color distribution of MPs in fish and sediment samples is similar to that in the water samples (Figure 3). MPs found in water samples of Lake Superior display a broader color spectrum compared to those found on beaches. The main colors of MPs in sediments and water samples are blue and pink [21]. In Finnish lakes, both water samples (87%) and sediment samples (48%) exhibit the highest concentration of transparent and white MPs at each sampling section. Other prevalent colors include gray and blue. Due to sediment samples retaining more diverse substances on the filters, transparent and white MPs may be more easily overlooked compared to more vivid colors. Previous studies on MPs have not shown a distinct color trend, with common colors ranging from blue to white and transparent [29].

MPs with different chemical compositions exhibit varying levels of toxicity and environmental hazards. Hence, when investigating MP pollution, scientists consider chemical composition as a crucial aspect. In Jingpo Lake, the predominant chemical compositions of MPs in water samples are Polyethylene (PE) (31.83%) and Polystyrene (PS) (25.48%), followed by polypropylene (PP) (17.56%), Polyamide (PA) (11.84%), polyethylene glycol terephthalate (PET) (6.71%),ethylene-vinyl acetate copolymer (EVA) (4.56%), and Polycarbonate (PC) (2.03%) in descending order (Figure 3). Other researchers have also identified PE, PP, and PS as the primary MP pollutants, followed by PP (17.56%). The proportion of MP chemical composition in fish and sediment samples resembles that in water samples. In Rawal Lake, Pakistan, MPs are mainly composed of PP, followed by low-density and high-density polyethylene (PE) [23]. The major components of MPs identified in China’s third-largest lake, Taihu Lake, were determined to be polyvinyl chloride and PE [17]. In Finnish lakes, among the 619 particles analyzed in water samples and 1194 particles in sediment samples, 89 and 210 particles were, respectively, identified as plastic. There are significant differences in the relative abundance of polymers detected in water and sediment samples. In the case of water samples, PP and Polyethersulfone (PES) are the most prevalent polymers, accounting for 33% and 29% of the MPs detected in sediment samples, respectively. PES covers 58% of the MPs detected, with PET being included in the count of PES. These findings align with the fact that polymers with densities significantly greater than 1 g/cm^3^, such as PES, tend to sediment more readily compared to polymers with lower densities. The polymer composition remains relatively consistent across sampling sections, but certain polymers, like polyamide (PA) and PS, are only present in a few samples. Despite PA being commonly used in textiles, this study detected only one type of PA fiber [30]. In Rewalsar Lake, the predominant polymers found in most MPs are polystyrene, PE, and PP polymers [20].

It is essential to emphasize the significance of conducting MP sampling across representative timeframes and spatial scales. Giving special consideration to conducting multiple sampling sessions throughout each year can provide a more comprehensive understanding of the sensitivity of lakes to MP loads and whether such loads are consistent [21]. Jingpo Lake’s MP contamination exhibits a certain degree of seasonality, with MP content in the water being higher during the summer (46.68%) compared to the spring (36.75%) and autumn (16.56%) (Figure 3). The proportions of MP content in fish and sediment samples during different seasons closely resemble those found in water samples. The area surrounding Jingpo Lake experiences both agricultural and summer tourism activities. During the summer months, the population increases, and agricultural practices involving plastic films and other plastic products become more prevalent. The combination of precipitation and surface runoff transports a significant quantity of MPs into the lake, potentially contributing to the elevated MP content in the water during the summer [23]. In Lake Superior, MP abundance per unit area was notably higher in samples of July 2018 (ranging from 18,000 to 40,000 n/km^2^) compared to May samples (ranging from 9000 to 11,000 n/km^2^) [18]. MP content within the digestive tracts of fish was found to be higher during the spring sampling period than in summer and autumn. This trend may be due to the fact that spring is the breeding season for fish, when increased consumption of planktonic organisms could lead to the ingestion and accumulation of MPs of similar sizes in the fish’s digestive tracts. Furthermore, MPs tend to remain undigested within the digestive tract, resulting in prolonged retention. During the autumn sampling period, MP content in water samples, fish bodies, and sediments was relatively lower. In Kumaraswamy Lake of Coimbatore, India, MP concentrations were found to be higher at the lake’s outlet during the monsoon season (12.41 ± 0.41 n/L) and pre-monsoon season (11.16 ± 0.47 n/L). In the central area of the lake, MP concentrations were approximately 10.16 ± 0.32 n/L (pre-monsoon), 11.33 ± 0.55 n/L (monsoon), 8.91 ± 0.57 n/L (post-monsoon), and 6.083 ± 1.003 n/L (summer). MPs in Xinghu Lake exhibited concentrations of 247 ± 120.6 during the dry season and 273.1 ± 353.7 during the wet season, which are quite consistent with the findings of this study [11]. Hassan et al.’s field experiment demonstrated that the lake’s hydrodynamics, influenced by changes in seasonal temperature, impacted the residence time and distribution of MPs in the mid-layer space during autumn. The retention time of MPs during lake turnover was significantly shorter than during the summer, likely due to turbulence in the water column. However, the understanding of MPs’ behavior in real lakes and their absorption probability by lake biota, including planktonic organisms, remains limited. Future research should quantitatively incorporate processes like heterogeneous aggregation and biofouling [31]. In the surface water samples of Manipal Lake in southwest India, the seasonal occurrence and distribution of MPs were observed. The concentration of MPs was higher during the monsoon season (0.423 n/L) compared to the post-monsoon period (0.117 n/L). This situation is attributed to inflows from rainwater drains connected to the lake and surface runoff during periods of heavy rainfall [22].

### 3.3. Analysis of Factors Influencing MP Abundance

**Figure 4:**
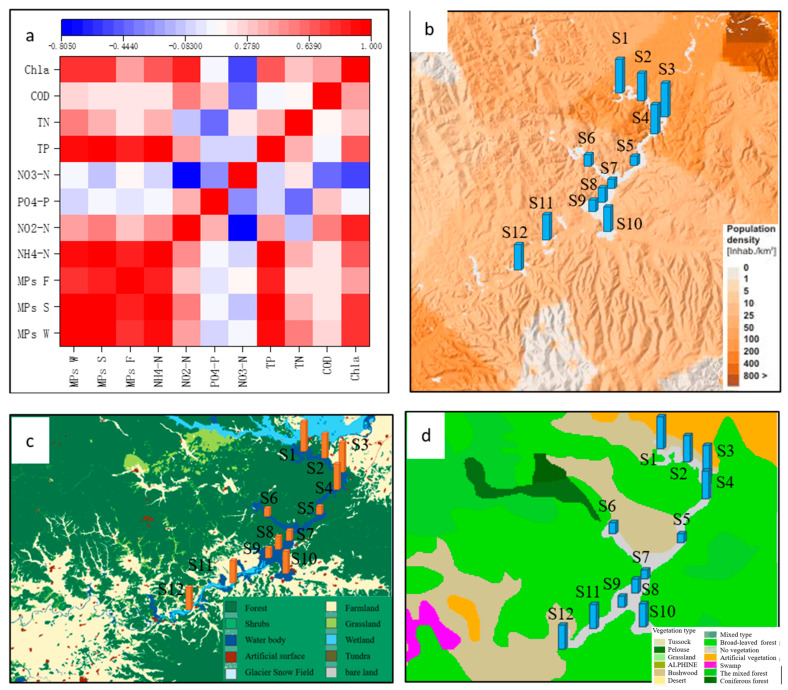
Factors affecting MPs in Jingpo Lake. (**a**): Correlation between MP content and other environmental physicochemical factors, MPs W−MP content in water, MPs S−MP content in sediments, MPs F−MP content in fish digestive tracts; (**b**): relationship between MP content and population density; (**c**): relationship between MP content and land use; (**d**): relationship between MP content and vegetation type.

MPs have been identified in samples from the surface water, sediments, and digestive tracts of fish at Jingpo Lake. Significant differences in MP concentrations exist among these three sample types, and notable spatial–temporal variations have been observed. These differences bear important ecological implications when assessing variations in MP loads across lakes and the potential impacts of MP pollution on these ecosystems. In this study, we place a strong emphasis on investigating these variations and their correlation with human activities (Figure 4).

The utilization of the Pearson correlation coefficient for assessing the spatial distribution of MPs in Jingpo Lake’s water, fish, and sediments predominantly indicates human activity influence. The results demonstrate a strong correlation between the content of MPs in water, fish, and sediments and the indicators of NH4-N, TP, and Chla present in the water (Figure 4a, α = 0.01). This suggests a homological relationship between MP pollution and the levels of NH4-N, TP, and Chla. Given that these three pollutants largely stem from domestic and waste pollution, it is plausible that MP contamination in Jingpo Lake originates from human sewage and waste. To validate this perspective, we scrutinized the impact of population density (Figure 4b), land usage (Figure 4c), and vegetation type (Figure 4d) on MP abundance. The key human activities in the region around Jingpo Lake encompass agriculture, tourism, recreational boating, industry, and fishing. Grounded on concentration levels in sediments and water, the infusion of MPs is more pronounced along the lake’s periphery. Sites S1–S4 are in close proximity to human settlements, tourist docks, hotels, and related reception facilities, while S2, S3, S10, S11, and S12 are closer to agricultural land. Consequently, higher MP loads were observed at S1, S2, S3, S4, S10, S11, and S12 (Figure 1). MPs have been detected in particle samples of surface water from all inland lakes in Minnesota, the USA. The study revealed the significant impact of land use and the level of lake development on MP loads within the lakes. Additionally, it highlighted the variability in MP loads and distribution within small inland lakes [21]. Findings from research conducted in a mid-altitude lake in the NW Himalayas indicated a clear correlation between the intensity of human activities and MP abundance. The results suggested that sources like domestic sewage, high-intensity tourism, and surface runoff from residential areas could constitute major area sources of MP pollution, posing substantial threats to lake ecosystems. A study published examined the degree of MP pollution in several lakes across Minnesota, considering varying levels of watershed development and population density [20]. The conclusions drawn from the MP concentrations detected in surface water and sediments, along with other environmental media, supported the hypothesis that watershed development and population density are critical factors influencing MP loads [21]. A research revealed that the Great Lakes exhibit substantial spatial variability in plastic pollution within collected samples. Among the three lakes studied, Lake Erie, with the highest population, likely accounts for the consistently elevated counts of plastic pollution observed in all eight samples collected from this lake compared to the other lakes. Despite Lake Superior having the lowest population density among the three, all five samples collected from it were situated closer to the shoreline (and thus closer to pollution sources) than those from Lake Huron. This spatial proximity elucidates why the average MP counts in Lake Superior samples surpassed those in Lake Huron [19]. Verónica et al. presented evidence suggesting that both natural factors and human activities influence the distribution and abundance of MPs. In their study, they examined the correlation between basin types and other geomorphic features (elevation, surrounding meadow area, basin type, and lake size), which could impact plastic pollution. Their findings indicated that the concentration of MPs was primarily associated with the extent of meadows encircling the lakes. This outcome implies an initial and significant linkage between the abundance of MPs and tourist activities. The variations in MP pollution among lakes are likely associated with materials brought by tourists, such as mountain gear, textiles, cosmetics, and recreational items, as evidenced by other lakes. Although the data do not establish a cause-and-effect relationship between tourists and MP quantities, it is noteworthy that lakes with 10 or more MPs per liter are situated along popular hiking routes [29]. These findings also reinforce the reliability of our research results within the Jingpo Lake basin. The diversity of MP polymers in Xinghu Lake also highlights the intricate and diverse nature of pollution sources in urban lakes subjected to intense human activities. Overall, activities like urban expansion and economic growth have indeed exerted a significant influence on the substantial accumulation of MPs in urban lakes [11]. The MP pollution in Vellayani Lake arises as a consequence of tourism and human interventions, encompassing activities such as fishing, the accumulation of waste near the lake shores, sediment weathering, and untreated sewage from these native regions. This MP contamination within lake environments has an impact on their prevalence in edible fish. The direct consumption of fish leads to the incorporation of MPs into the human body [9]. The outcomes obtained from assessing MPs in lakes across the Nevada Mountains similarly lend support to the conclusions drawn in this study [27].

### 3.4. Diversity Analysis of MP Pollution

Whether conducting assessments of MP pollution risks or tracing pollution sources, addressing the diversity of MP pollution is a primary concern. To this end, scientists have developed various methods for assessing the diversity of MP pollution. Sun et al. were the pioneers in using the Shannon–Wiener Index to analyze the diversity of MP chemical compositions in regions like the Yellow Sea in China. The results demonstrated that the Shannon–Wiener Index is a robust approach for deciphering the sources and pollution patterns of MP contaminants [2,31]. This method has also been adopted and widely applied by numerous researchers in their studies. The diversity index (Shannon–Weaver Index) combines the richness and evenness of species or types within an ecosystem, enabling the quantification of the uncertainty in the system’s structure. In this context, we utilize this index to calculate the MP diversity for each sampling section within the four surveyed regions. The formula is as follows:

(1) $$H^{\prime }=-\sum_{i=1}^{n}PiLnPi$$

where *P_i_* represents the proportion of category *i* in the total sample, *N* is the number of categories, and *P_i_* = *N_i_*/*N*.

**Figure 5:**
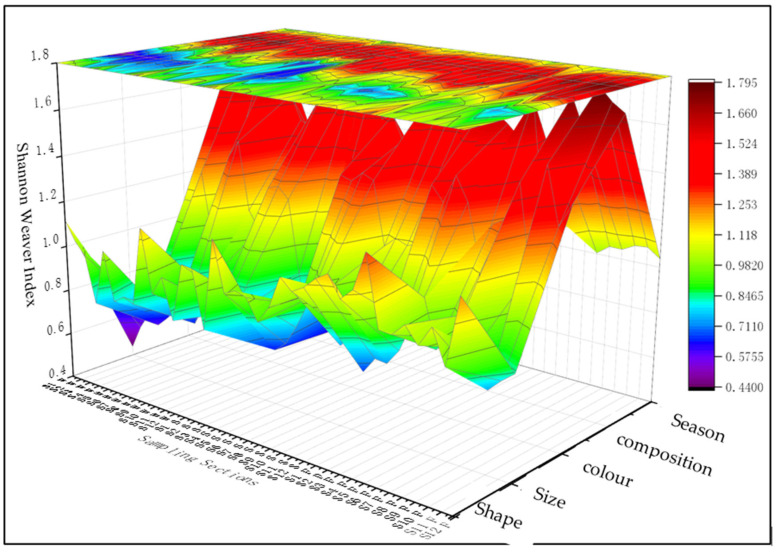
Diversity of MP pollution in Jingpo Lake. S1W–S12W: MPs in water; S1S–S12S: MPs in sediments; S1F–S12F: MPs in fish digestive tracts.

By substituting the data on MP pollution from water samples of Jingpo Lake, sediment samples, and fish digestive tract samples into the above formula, the MP diversity of Jingpo Lake is obtained (Figure 5). Among them, the chemical composition diversity of MP pollution, as measured by the Shannon–Wiener Index, is the highest, while the diversity index for size has the narrowest range, specifically chemical composition (1.23–1.79) > color (0.59–1.54) > shape (0.78–1.30) > season (0.83–1.10) > size (0.44–1.01).

Scientists have studied the impact of the 2019 coronavirus disease (COVID-19) pandemic on the occurrence of microplastic diversity in the aquatic environment and found that the amount of plastic released into the aquatic environment by different aquatic sub-regions of the Taihu Lake Lake basin showed a downward trend during the outbreak from 2019 to 2021 [2].

### 3.5. Ecological Risk Assessment

When assessing the potential risks of MPs in aquatic environments, it is essential to consider risk assessment models that incorporate both MP polymer toxicity and MP abundance. A study conducted byintroduced polymer risk indices, revealing that MPs in Jingpo Lake predominantly consist of substances with low toxicity, as indicated by air toxicity indices below 10 (Table 2) [32]. To evaluate the environmental risks of MP pollution, three models are adopted in this study: the polymer hazard index (PHI), pollution load index (PLI), and potential ecological risk index (PERI), which were applied to assess MP pollution in water samples [20,33,34]. The specific formulas for these models are as follows:

*PHI* = ∑*P*_*n*_ × *S*_*n*_ (2)

where *PHI* represents the polymer hazard index, *P_n_* represents the percentage of each polymer at each sampling section, and *S_n_* denotes the toxicity score of the polymer.

The PLI is employed to assess the MP load at each site. It is correlated with the abundance of MPs, and its calculation formula is as follows:

*CF* = *C*/*C*_0_ (3)

(4) $${PLI}_{n}=\sqrt{CF}$$

where *CF* represents the degree of MP contamination; *C* signifies the MP abundance at each sampling section; *C*_0_ stands for the background value, i.e., the level of MP abundance in the absence of contamination; *PLI* corresponds to the MP load index for a specific site; and *n* denotes the total number of sites. To facilitate the comparison of MP pollution risk across various regions, it is essential to determine a specific value for *C*_0_. Due to the scarcity of available background data, this study opted for the lowest recorded MP abundance (106.7 n/L) as the background value. The classification standards for polymer toxicity scores and polymer risk indices are presented in Table 3. Both the PHI and PLI are utilized to assess the potential risks of MPs in Jingpo Lake.

In addition to the PHI and PLI, the PERI primarily focuses on the quantitative analysis of pollutants. The PERI is a crucial method for assessing the potential ecological risks of pollutants in environmental media and finds widespread use in investigating various pollutants, such as heavy metals. The PERI can be considered a comprehensive assessment approach that combines both the PHI and PLI. Therefore, in this study, an enhanced version of the traditional PERI, as proposed by some researchers was adopted as a reference [35]. The formulas are as follows:

(5) $$C_{f}^{i}=C_{i}{/C}_{n}^{i}$$

(6) $$T_{r}^{i}=\sum_{i=1}^{n}\frac{P_{n}}{C^{i}}\times S_{n}$$

(7) $$PERI=T_{r}^{i}\times C_{f}^{i}$$

where *C_i_* and ${\;C}_{n}^{i}$ represent the concentrations of observed plastic polymers and background concentrations, respectively. The toxic coefficient ($T_{r}^{i}$) signifies the level of toxicity and biological sensitivity. This coefficient is calculated as the sum of the percentage of specific polymers in the total sample (*P_n_*/*C_i_*) multiplied by the hazard score (*S_n_*) of the plastic polymers. The assessment outcomes are classified into categories such as minor, medium, high, danger, and extreme danger based on the magnitude of the index (Table 3).

**Figure 6:**
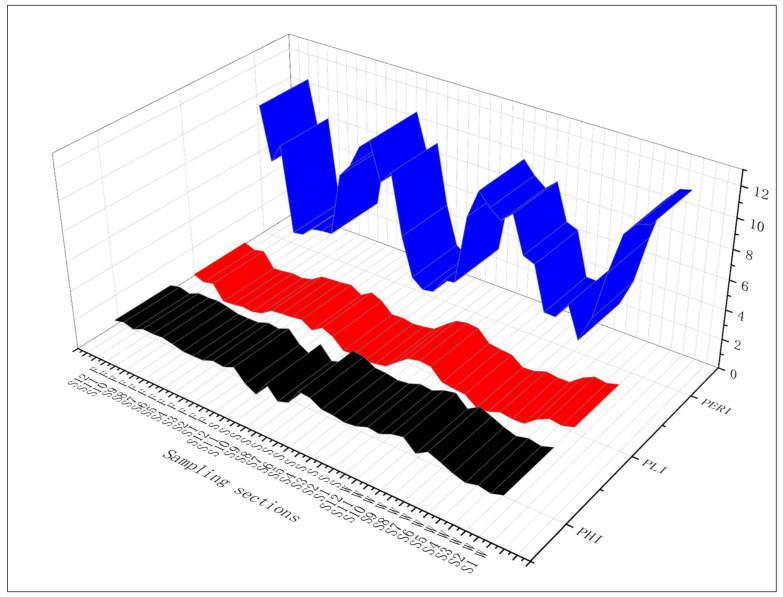
Risk assessment of MPs in Jingpo Lake. S1W–S12W—MPs in water; S1S–S12S—MPs in sediment; S1F–S12F—MPs in fish digestive tracts.

Twelve sampling sections of Jingpo Lake are evaluated according to the criteria provided in Table 3. The risk category of the PHI, PLI, and PERI are I, I, and minor, respectively (Figure 6).

The values of the three risk assessment indicators are relatively low, which is attributed to the low toxicity of the MPs distributed within the Jingpo Lake basin. However, the results of the risk assessment demonstrate certain patterns. Sampling sections 1, 2, 3, 4, 10, 11, and 12 exhibit higher risk indices, while the remaining five sampling sections present lower risks. This coherence is consistent with the results of the correlation analysis between the population, cultivated land distribution, and MP pollution at the sampling sections (Figure 4b,c). Some study on Rewalsar Lake indicated that the concentration of certain MPs and associated compounds exceeded recommended environmental risk thresholds. The outcomes of this study underscore the necessity for implementing suitable waste management measures in the region to curtail the influx of these pollutants into the ecosystem. Furthermore, it is imperative to enhance monitoring to regulate the dissemination of emerging pollutants and their influence on the biota [20]. As an urban lake in China, MPs in Xinghu Lake are mainly composed of PET, RY, PP, and PE. The ecological risk assessed using the PERI index is categorized as Level III [11]. Similarly, for the urban river East Lake, the PLI in water samples is 0.66, and the sediment PLI is 1.89, indicating that over half of the sampling sections exhibit risks ranging from danger to extreme danger. The predominant chemical composition of MPs includes PET, PP, PE, and PVC [5]. In southern India, Lake Manipal’s MPs are primarily composed of PET and CL. The risk level of MPs in the lake exhibits seasonal variation. During the monsoon period, the PLI of Lake Manipal is 1.62, which slightly decreases to 1.39 after the monsoon. PLI values consistently remain below 10, indicating a risk level of 1. For the Kodaikanalan Lake (freshwater) in India, the average PLI in sediments is 1.33, which can be classified as risk level I (<10), while the PHI is classified as risk level V (>1000) due to the presence of MPs made from PEU and PS. However, these current levels represent relatively minor ecological risks [22]. In the case of Kumaraswamy Lake in Coimbatore, India, during the monsoon season, the PLI for MP contamination is higher at the lake’s outlet (1.15) and center (1.36) areas compared to the inlet (1.68) [7]. Some researchers evaluated the risk index of MP pollution in Vellayani Lake, Kerala, in 2023. The results indicate the PLI of sediment: ND-3.87, PERI: ND-536, water PLI: ND-3.32, PERI: ND-516. The main components of MPs in the lake are PY, PP, PE, etc. [9].

## 4. Conclusions

This is the first recorded instance of MP pollution in Jingpo Lake. Most studies on MP pollutants in water bodies have primarily focused on oceans, and research on freshwater MPs has mainly centered on how MPs enter the oceans through waterways. However, remote mountain lakes like Jingpo Lake, which are situated far from major population and industrial centers with limited transportation access, have traditionally been regarded as more pristine environments. These lakes are shielded from the industrial pollution commonly found in urban lakes, making them well suited for understanding the influence of factors such as agriculture and population on the abundance of MPs in aquatic ecosystems. This study contributes to a more comprehensive understanding of the global distribution of plastic pollution. Additionally, this study indicates that human activities, including gatherings and tourism, can significantly impact the accumulation of MPs in these natural environments. The abundance of MPs in the surface water, sediments, and fish digestive tracts of Jingpo Lake falls within a medium range, with their spatial and temporal distribution influenced by factors such as human settlements, agricultural practices, and tourism activities. The diversity of MP pollution in Jingpo Lake is reflected in various aspects. Notably, the Shannon–Wiener Index demonstrates the highest diversity in terms of the chemical composition of MP pollutants, whereas the diversity index for size exhibits the narrowest range. Specifically, the diversity ranking is as follows: chemical composition (1.23–1.79) > color (0.59–1.54) > shape (0.78–1.30) > season (0.83–1.10) > size (0.44–1.01). The correlation analysis underscores that the primary source of MPs is attributed to human activities and waste. When assessing the ecological risk associated with MPs in the lake’s surface water, fish, and sediments, the risk category of the PHI, PLI, and PERI is classified as I, I, and minor, respectively. The values of these three risk assessment indicators are relatively low, aligning with the correlation analysis outcomes regarding the relationships between population, land use, and MP pollution in the sampling sections. Factors like land use along the lake shore and population density exert an influence on the MP loads in water, sediments, and fish. In Jingpo Lake, instances of anomalies among animals have been observed, including combinations of nematode infections, skin lesions, and prolapsed cloacae. Whether these anomalies directly stem from the ingestion of MP particles by amphibians and reptiles in Jingpo Lake remains uncertain. Therefore, the foundational MP data offered by this study and comparable research are of paramount importance, as they might potentially facilitate future analyses of the potential correlation between MP pollution and the occurrence of diseases in both human and other biological populations.

## Figures and Tables

**Table 2:** The value of the PHI.

Polymer Type	Toxicity Score of Polymers
PE	1
PP	1
PS	6
PA	1
PET	4
PC	1
EVA	3

The data for toxicity score are from Delilah et al., 2011 [33].

**Table 3:** Classification criteria for MP polymer risk indices (PHI, PLI, and PERI).

PHI	Risk Category	PLI	Risk Category	PERI	Risk Category
0–10	I	<10	I	<150	Minor
10–100	II	10–20	II	150–300	Medium
100–1000	III	20–30	III	300–600	High
1000–1500	IV	>30	IV	600–1200	Danger
>1500	V	-	-	>1200	Extreme Danger

## Data Availability

The original contributions presented in this study are included in the article. Further inquiries can be directed to the corresponding authors.
